# Proteomic Landscape of Prostate Cancer: The View Provided by Quantitative Proteomics, Integrative Analyses, and Protein Interactomes

**DOI:** 10.3390/cancers13194829

**Published:** 2021-09-27

**Authors:** Nithin Sadeesh, Mauro Scaravilli, Leena Latonen

**Affiliations:** Institute of Biomedicine, University of Eastern Finland, FI-70210 Kuopio, Finland; nithin.sadeesh@uef.fi (N.S.); mauro.scaravilli@uef.fi (M.S.)

**Keywords:** androgen receptor (AR), prostate cancer, castration-resistant prostate cancer (CRPC), proteomics, integrative analyses, protein interactomes

## Abstract

**Simple Summary:**

Recent methodological developments have enabled studying proteins on a large scale in a quantitative fashion, which helps in building comprehensive views of specific molecular settings. These proteomics approaches, and combining their information to genomics and transcriptomics, have revealed that not all genetic and transcriptomic aberrations in prostate cancer are translated to the proteome. This makes it important to understand which ones are translated. Here, we review recent large-scale proteomics studies on clinical prostate cancer and prostate cancer models which help us understand how prostate cancer develops and evades current drug treatments.

**Abstract:**

Prostate cancer is the second most frequent cancer of men worldwide. While the genetic landscapes and heterogeneity of prostate cancer are relatively well-known already, methodological developments now allow for studying basic and dynamic proteomes on a large scale and in a quantitative fashion. This aids in revealing the functional output of cancer genomes. It has become evident that not all aberrations at the genetic and transcriptional level are translated to the proteome. In addition, the proteomic level contains heterogeneity, which increases as the cancer progresses from primary prostate cancer (PCa) to metastatic and castration-resistant prostate cancer (CRPC). While multiple aspects of prostate adenocarcinoma proteomes have been studied, less is known about proteomes of neuroendocrine prostate cancer (NEPC). In this review, we summarize recent developments in prostate cancer proteomics, concentrating on the proteomic landscapes of clinical prostate cancer, cell line and mouse model proteomes interrogating prostate cancer-relevant signaling and alterations, and key prostate cancer regulator interactomes, such as those of the androgen receptor (AR). Compared to genomic and transcriptomic analyses, the view provided by proteomics brings forward changes in prostate cancer metabolism, post-transcriptional RNA regulation, and post-translational protein regulatory pathways, requiring the full attention of studies in the future.

## 1. Introduction

Prostate cancer is the second most frequent cancer and the fifth cause of cancer-related death among men worldwide [1]. Most men diagnosed with prostate cancer present with localized disease confined within the prostate for which surgery and radiation therapy represent effective treatment strategies [2,3]. Recurrence after surgery and/or radiation often indicates that the cancer has spread outside the primary site and has progressed to metastatic disease [4]. As prostate carcinogenesis is highly dependent on androgens, androgen deprivation therapy (ADT) represents the first-line treatment for metastatic disease. However, 80% of cases treated with ADT will eventually become unresponsive and develop a castration-resistant prostate cancer (CRPC), which remains uncurable [5,6]. Therefore, there is a need for more effective therapeutic strategies for the advanced disease, as well as reliable biomarkers to use at the time of diagnosis for identification of the cases that are likely to progress.

Tumor grade, as determined by the Gleason score (GS), is one of the most important predictors of clinical outcome of primary prostate cancer (PCa). GS is formed by the sum of the two most prevailing histopathological patterns in the tumor, as graded between 1 and 5 (G1–5). Patients with a low GS (≤6) are considered to have low-risk disease and, therefore, to be suitable for active surveillance. Patients with a high GS (8–10) are considered to have high-risk disease and are, in general, actively treated. The expected outcomes for patients with GS 7, however, are less clear. This watershed grade can be further divided based on representing either a mostly well-differentiated cancer with a lesser component of more poorly differentiated cancer (G3 + 4), or a mostly poorly differentiated cancer with a small component of well-differentiated cancer (G4 + 3). A revised version of the Gleason scoring system, taking into account the challenges with GS7, has been introduced by the International Society of Urological Pathology (ISUP). In the ISUP system, PCa is divided into five grades: grade 1 (GS ≤ 6), grade 2 (GS = 7, with a primary and secondary pattern of 3 + 4), grade 3 (GS = 7 with a primary and secondary pattern of 4 + 3), grade 4 (GS = 8), and grade 5 (GS = 9–10) [7]. The ISUP grades correlate with patient 5-year survival rate, decreasing from approximately 95% at grade 1 to 35% at grade 5 [8]. However, tumor heterogeneity and subjective variations in biopsy sampling and interpretation of the GS impact the treatment decisions and patient outcome [9].

AR is a ligand-dependent transcription factor that plays a critical role in both normal development of the male phenotype and development and progression of prostate cancer. AR binds androgens with high affinity in the cytoplasm and is subsequently translocated to the nucleus, where it binds specific androgen-response elements (AREs) in the genome and activates the expression of androgen-regulated target genes [10]. In prostate cancer cells, the AR is responsible for activation of genes involved in cell proliferation and tumor progression [11]. Despite low androgen levels resulting from ADT, CRPC maintains its dependence on AR activity. This can be achieved via several molecular mechanisms, such as AR overexpression, AR mutation, or expression of constitutively active AR splice variants [12].

The most common genetic alteration in prostate cancer consists of a chromosomal rearrangement between the androgen-regulated gene *TMPRSS2* and the ETS transcription factor *ERG* [13]. This genetic fusion occurs in approximately 50% of prostate cancer cases and results in androgen-driven overexpression of ERG. The consequent upregulation in transcriptional activity of ERG is associated with prostate tumorigenesis and is responsible for increased invasiveness of prostate cancer cells in metastatic disease [14,15]. Moreover, the *TMPRSS2-ERG* fusion event is associated with overexpression of the oncogene MYC in prostate cancer cells, which further contributes to promote cell growth and proliferation [16]. Chromosomal region 8q24, the site harboring the gene coding for transcription factor c-MYC, is often amplified in prostate cancer [17]. c-MYC overexpression modulates protein synthesis, cell cycle, and metabolism. Another common alteration in prostate cancer is the inactivation of the tumor-suppressor gene *PTEN*, which is found in approximately 20% of PCa and up to 50% of advanced tumors and is often caused by gene deletion or mutation [18,19]. The phosphatidylinositol 3-kinase (PI3K) pathway is implicated in both prostate carcinogenesis and castration resistance [20]. PTEN functions as an antagonist of the PI3K pathway, which results in downstream activation of AKT, leading to regulation of target genes involved in cell proliferation, apoptosis, differentiation, and invasion [21]. Alterations of tumor suppressors p53 and RB also accumulate with disease progression [22]. More recently, several genome-wide sequencing studies performed on clinical samples of prostate tumors revealed multiple recurrent alterations that involve both coding and non-coding genes that were not previously implicated in prostate cancer tumorigenesis, such as those of *NCOA2*, *FOXA1*, *SPOP,* and *IDH1* [23,24,25,26]. These novel targets function as oncogenes or tumor suppressors and allow for a classification of prostate cancers in distinct classes. Moreover, the availability of significantly larger clinical sample cohorts has recently provided the opportunity to characterize genetic alterations that are present at lower frequencies, revealing a substantial inter-patient heterogeneity of genetic changes, where many genes that drive prostate cancer are mutated in less than 3% of cases [27]. Genetic aspects and their clinical correlates have been extensively discussed previously [28,29,30].

A demand exists for identification of potential biomarkers for patient stratification according to prognostic risks to avoid over- and undertreatment of patients. To develop more efficient treatments for lethal forms of prostate cancer and to prevent disease relapse, the mechanisms involved in cancer development and progression need to be better understood. While the genetic alterations shed light on events behind cancer development and tumor heterogeneity, tailoring treatments based on the genetic “long tail of drivers in prostate cancer” [27] is challenging and seems mostly unfeasible. Moreover, many of the mutations likely signal through shared major pathways and affect similar critical cellular functions. Considering further that most success stories in cancer therapeutics are still based on targeting protein functions [31], we need a deeper understanding of prostate cancer molecular events at the functional levels beyond the genome and the transcriptome. The proteome, with all its post-translationally modified versions, and the metabolome are invaluable levels of information we need to better understand the translations of cancer genomes in each prevailing context, and to resolve how we can target the critical events driving cancer development and evolution. It has already been shown for several cancers that the proteome does not duly replicate the cancer transcriptome [32,33,34] and, as discussed below, this also seems true for prostate cancer. Thus, many alterations at the nucleotide level are left without an effect at the level of the proteome. Hence, understanding the cancer proteome becomes essential to complement the molecular information provided by cancer genomes and transcriptomes.

**Figure 1:**
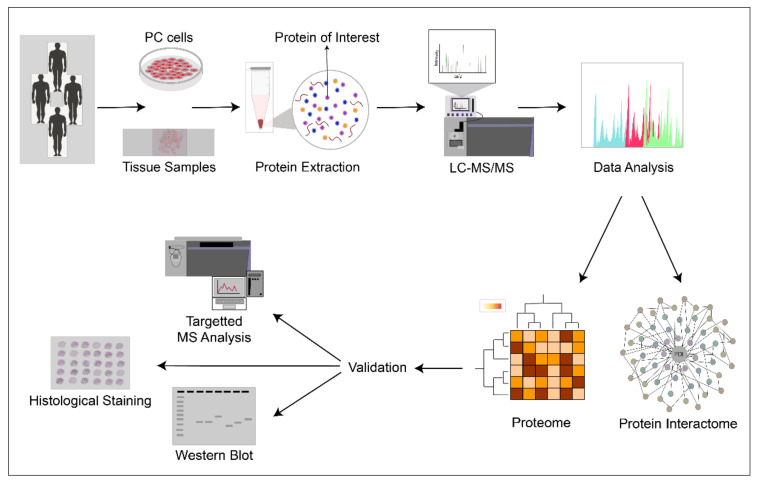
Prostate cancer proteomics. A typical outline of current studies involving patient tumor tissue samples and prostate cancer (PC) cell lines which are subjected to protein extraction and analyzed by quantitative mass spectrometry (LC-MS/MS) either for full proteomes or specific sub-proteomes, depending on sample preparation. Typically, datasets and identified proteins of interest are validated using alternative methods and additional samples.

Here, we review what is currently known about mechanisms of prostate cancer based on recent developments with prostate cancer proteomics. We focus on proteomes in prostate tumors and cells with recent, quantitative, large-scale mass spectrometry (MS)-based analytics, including specific interactomes of central prostate cancer factors (Figure 1). While two-dimensional gel electrophoresis (2-DE) and earlier MS-based efforts have produced a massive amount of important information and been very useful in identifying individual differentially expressed proteins (DEPs) (reviewed in [35]), their output level is usually at identification of a couple of dozens or hundreds of proteins. Improvements in sample preparation, sensitivity of mass analyzers, and development in computational analysis methods now allow quantitation of almost full proteomes with thousands of reliably quantified proteins with high reproducibility. Methods improving extraction of proteins from formalin-fixed, paraffin-embedded (FFPE) tissue have allowed utilization of archived tumor material and, on the other hand, the development of interaction assessment techniques have enabled steps towards increased understanding of dynamic prostate cancer proteomes. Rather than focusing on identification of individual DEPs, here, we review the broad-scale view and pathway information provided by the studies producing the large-scale and dynamic proteomes. Proteomics in biomarker discovery aiming for improved disease management of prostate cancer have recently been excellently reviewed by others [36,37,38]. Proteomic efforts to identify biomarkers from body fluids and vesicles have also been reviewed elsewhere [39,40,41,42,43].

## 2. Proteomes of Clinical Prostate Cancer Samples

Several highly informative large-scale proteomics analyses have been published from primary prostate tumors (PCa) as well as advanced local CRPC and distal metastases (Table 1). All studies so far assess sporadic prostate cancer cases. Although the overall number of identified proteins is often much higher, in most of the current studies, the high-confidence proteome spanning all samples in a sample set comprise of a thousand to a few thousand proteins. Most of the studies analyze a relatively small sample number, reflecting the fact that these studies are still not trivial to perform for large numbers of tumor samples. Many studies utilize freshly frozen tissue material, but also FFPE samples have been successfully assessed, and optimized protocols for FFPE samples are being developed [44,45]. The datasets generally reflect the methodological bias of identification correlating with abundance of proteins and, thus, the data still do not often reflect the full proteome of clinical prostate cancer. However, compared to the previous views provided by 2-DE-based approaches identifying proteins in the order of hundreds and DEPs often in the range of a few dozen, the recent large-scale studies utilizing the enhanced MS/MS approaches provide remarkable new insight into proteomic changes throughout the development and progression of prostate cancer. Further, they reveal the potential of using proteomic data to stratify patients and develop new therapeutic strategies. Especially, the first studies integrating genomics, epigenomics, transcriptomics, and proteomics of prostate cancer have provided novel insights and views to the disease, helping significantly in identifying the relevant changes from the sea of cancer alterations. Many studies identify top candidates, suggest novel markers and subgroupings, and verify alterations in expression of certain proteins. Due to broad variance in these proteins, the readers are directed to the original studies (Table 1) for lists of individual proteins and findings in particular set-ups.

### 2.1. Large-Scale Proteomics of Primary Cancer of the Prostate

Quantitative proteomics studies comparing primary tumors to benign tissue (Table 1) describe a number of DEPs which have been identified between PCa and non-malignant tissue from the same patient [59], PCa and benign prostatic hyperplasia (BPH) [50,53,56], low-risk and high-risk prostate cancer groups [46], low- and high-grade prostate cancer [51], different ISUP grades [50], and even laser-capture microdissection (LCM)-isolated cellular fractions, such as epithelial cells of different Gleason grade [48] or ERG status [60]. Although many of these studies find alterations in expression levels of several proteins previously well-known to be aberrantly expressed in prostate cancer, significant heterogeneity exists in the identity of the DEPs identified. This may result from several factors. Firstly, the proteomic datasets often do not represent the full proteome, leaving many proteins excluded from the analysis. Secondly, to understand molecular changes taking place in tumorigenesis, PCa is typically compared to benign tissue. In proteomic studies as well as many other types of assessments, the type of benign tissue varies, resulting in heterogeneity between what is considered “normal” and thus affecting what is interpreted as “change”. Thirdly, there is variation in the grades included in the studies assessing PCa. Additionally, differences of tissue preservation methods, quantification methods, relative proportion of cancerous tissue and stroma in the samples, and cutoffs used for identifying DEPs are likely to affect the results. Furthermore, as the number of samples in many studies is low, the significance power to identify changes is also low, especially as inter-patient heterogeneity of tumors exist. Interestingly, intra-patient heterogeneity has been reported to be higher in proteomes of benign samples compared to primary cancer [49,50]. The findings by Zhou et al. [51] suggest that, compared with normal prostate, most protein expression level changes that are statistically significant in high-grade prostate cancer were already present in low-grade prostate cancer samples, although their extent was less pronounced.

#### 2.1.1. Functional Pathways’ Surfacing Based on Proteomics Studies of Primary Prostate Cancer

Despite that the lists of DEPs overlap only partly between studies published thus far, the large-scale proteomics studies have reported remarkably systematic changes in the functional pathways altered during development of prostate cancer. Metabolic pathways are the most consistently reported as significantly altered in proteomes of PCa [46,47,50,53,56,57,59]. Especially fatty acid synthesis is upregulated in PCa, and alterations in the tricarboxylic acid cycle (TCA cycle, Krebs cycle, citric acid cycle), glycolysis, oxidative phosphorylation, and amino acid metabolism pathways are often detected. Changes in metabolic pathways can be detected even between different grades of primary tumors (GS3 vs. GS4 epithelium) [48], and primary tumors from metastasizing cancers are enriched in lipid metabolic processes compared to non-metastasizing primaries [57]. Primary tumor samples show higher levels of mitochondrial proteins [46], and mitochondrial complex I is upregulated, especially in high-grade primary tumors [51].

The next most detected alterations are linked to cell adhesion and cytoskeletal events. Cell adhesion-linked proteins and processes are found consistently downregulated in PCa tissue [50,56,59], especially for integrins and the integrin-linked kinase pathway [51,56]. For example, adhesion was found to be the most altered process in ISUP grades 1–4 compared to BPH [50], and metastasizing primary tumors have decreased adhesion processes compared to non-metastasizing primaries [57]. Cytoskeletal processes and the actin network are altered in PCa vs. BPH [53,56].

RNA processing and protein turnover have received more attention via proteomics than based on earlier sequencing studies. Spliceosome is upregulated in low-grade primary tumors [51], RNA processing and mRNA splicing in G3 vs. G4 epithelium [48], RNA binding proteins, translation, and rRNA processing are upregulated in tumors compared to normal tissue [59], and nucleic acid binding proteins are upregulated in PCa vs. BPH [53]. Translation- and protein degradation-related pathways, such as protein synthesis, ribosomal biogenesis, and protein secretion, are dysregulated in primary tumors compared to benign tissue [46,56]. Protein folding is dysregulated in ISUP grade 5 compared to BPH [50], protein processing in PCa vs. BPH [53], and unfolded protein response (UPR) and protein ubiquitination in PCa vs. BPH [56]. Deregulation of protein turnover pathways is associated with induction of MYC targets and endoplasmic reticulum (ER) proteins in PCa vs. BPH [53].

Other significantly altered pathways detected in large-scale proteomics of PCa include apoptosis, which is downregulated in cancer compared to normal cells [51], and in ISUP grade 5 compared to BPH [50]. DNA damage and telomere extension pathways are upregulated in PCa vs. BPH [56]. Of the signaling pathways, especially PI3K/AKT/mTOR, RXR-related, and GTPase signaling are altered in PCa vs. BPH [53,56]. In PCa, vesicle traffic is altered compared to BPH [56]. Interestingly, ETS fusions were associated with alterations in pathways governing intra- and extra-cellular vesicles and lysosomal genes [52]. Of other extracellular events, platelet aggregation and complement activation seem to be downregulated in prostate cancer primary tumors compared to normal tissue [59]. PCa also shows decreased expression of many ECM proteins [51,59]. This, and the decrease in proteins related to muscle contraction [59], may reflect the decreased amount of stroma in tumor samples compared to benign samples. For example, Zhou et al. [51] reported that the total number of downregulated proteins is larger than that of upregulated proteins, partly reflecting decreased amounts of stromal cells and expression therein. In their protein network analysis, the upregulated networks were mostly cytoplasmic or nuclear, and the downregulated networks to a large extent localized in the plasma membrane and cytoplasm [51].

Tan et al. [60] specifically studied the proteome linked to the ERG status of prostate tumors. They identified tumor cells positive or negative for ERG expression by immunohistochemistry (IHC) staining, followed by isolation of negative and positive cell populations with microdissection (LCM). Benign cells distant to tumor foci and tumor cells were isolated from the same FFPE tissue sections of five patients with Gleason grade 3 + 3 or 3 + 4, and the analysis was performed on pooled samples (of two ERG+ tumors, of three ERG− tumors, and of five benign specimens): 1196 DEPs were identified, of which 518 and 500 were unique to ERG+ and ERG− tumor cells, respectively. Pathway analysis of DEPs revealed enrichment of pathways promoting cell survival, cell growth, and protein synthesis (AKT and mTOR pathways), as well as pathways regulating cell shape, cytoskeleton, and motility (PAK and CDC42 pathways) in the ERG+ tumors. Pathways enriched in ERG− tumors regulate degradation of proteins (proteasome pathway), actin remodeling and cell migration (calpain and CDC42-RAC pathways), MAPK pathway activation (GPCR pathway), and prevention of oxidative damage (redox pathway) [60].

#### 2.1.2. Utility of Large-Scale Proteomics in Patient Stratification and Personalized Medicine

The identification of numerous DEPs in the large-scale (Table 1) and other recent quantitative proteomics studies revealed several potential individual markers for prostate cancer. For example, pro-neuropeptide Y (pro-NPY) was identified as a novel prognostic biomarker in early prostate cancer [46]. N-acylethanolamine acid amidase and protein tyrosine kinase 7 were shown to be markers for aggressive prostate cancer [54]. Pyruvate dehydrogenase kinase 4 (PDK4) could also be a potential prognostic target in prostate cancer [61]. High expression of MVP was found to be associated with poorer survival in PCa patients [62]. Myers et al. compared age-, stage-, and Gleason score-matched prostate tumor specimens from African American men with higher prostate cancer incidence and mortality to Caucasian American men, but were unable to detect statistically significant DEPs. However, over-representation and pathway enrichment analysis showed that cytoskeletal, ECM, and adhesion pathways as well as basement membrane and coagulation proteins were over-represented in tumors from African American patients.

Since the power of individual markers over the current gold standard prostate-specific antigen (PSA) has so far been limited, larger panels of DEPs have been suggested for patient stratification and prognostics. So far, mostly due to the low numbers of samples in the datasets, identification of marker panels and patient subtypes based on tumor tissue proteomics are rare. Kawahara et al. [50] suggested an 11-protein panel (composed of proteins IGKV3D-20, RNASET2, TACC2, ANXA7, LMOD1, PRCP, GYG1, NDUFV1, H1FX, APOBEC3C, and CTSZ) to further assess for potential use to stratify patients to low and high prostate cancer grades. Sinha et al. [52] identified five clusters of patients according to the top 25% most variable proteins (n = 1800) in their proteomics data. Two of these patient clusters were associated with an increased rate of biochemical recurrence. These subtypes were independent of the AR activity signatures of the tumors, as expected for treatment-naïve, hormone-sensitive tumors that the sample panel contained [52]. The abundance of specific proteins was found to associate with clinical phenotypes, for example, tumor size (8 proteins) and presence of aggressive intraductal carcinoma/cribriform architecture (7 proteins, including decreased expression of tumor suppressor PTEN). Further studies are required to evaluate the potential usefulness of these panels and clusters in patient stratification.

Proteomes of specific protein modifications are also queried with respect to patient stratification, as rapid advancement of targeted MS platforms could be utilized in clinical settings [63,64,65]. Separation of aggressive from non-aggressive PCa based on N-glycoproteome seems moderate so far [54]. Phosphoproteomics, on the other hand, seems potentially beneficial for oncologists in therapy decisions and management of individual cancers via personalized identification of biomarkers and drug targets [63,64,66]. AR affects the expression of several kinases and phosphatases, and results from phoshoproteome studies indicate active signaling in prostate cancer and CRPC by several kinases that might represent viable therapeutic targets, such as YAP1 and PAK2 (reviewed in [67]). On the other hand, ADT induces activation of certain kinase pathways, such as that of PI3K/AKT [67]

#### 2.1.3. Proteomics in Assessing Heterogeneity in Prostate Cancer

Prostate cancer is a heterogeneous, multifocal disease. Heterogeneity in cellular composition within a given tumor, between tumor areas and foci in the same patient, and between patients, are forms of tumor heterogeneity linked to prostate cancer prognosis as well as differences in responses and resistance to treatment (recently reviewed in [30]). For example, AR, PTEN, and ERG expression can vary between different regions within the same prostate carcinoma [68,69]. Thus, it remains a challenge to optimize clinical decisions based on single biopsies [70]. Further, increased understanding of the extent of heterogeneity and its clinical significance is required, making assessment of spatial heterogeneity of importance. High-throughput IHC, which is based on antibody staining, is an interesting technique that has already been performed on tissue sections [71]. This type of data is, however, currently semiquantitative and very much limited by the availability of antibodies suitable for the method. Another promising technology is single-cell proteomics by mass cytometry, which allows for quantitation of protein levels in thousands of individual cells. However, only tens of proteins per sample can currently be measured with this technique [72].

Proteomics can also be utilized in studies of tumor heterogeneity. While most studies use bulk samples extracted from tumor areas that are confirmed to contain mostly cancerous tissue, these datasets also always contain proteins originating from non-tumor cells, such as stromal and vascular cells. Thus, assessment of intra-tumor heterogeneity at a cellular level requires specialized methods to distinguish between signals originating from different cells within the tumor. By assessing grade differences and tumor microenvironment via label-free quantitative proteomics of LCM-isolated epithelial and stromal cells from Gleason grade 3 vs. 4 tumors (n = 4 for each), Staunton et al. [48] identified changes in immune response and cell-to-cell signaling between G4 and G3 stroma. Another way to assess intra-tumor heterogeneity is sampling the same prostate tumor from several areas and comparing their proteomic profiles. In an interesting study by Guo et al. [49], a total of 60 samples derived from 3 patients (12 (6 normal, 6 acinar) from patients 1 and 2, and 36 (12 normal, 12 acinar, 12 ductal) from patient 3) was used to study tissue spatial heterogeneity in prostate cancer [49]. Spatial and cell type intra-tissue variability was found to be the same extent as inter-patient variability, and protein variances between patients, between tissue type (benign and prostate cancer), and within tissue were significantly correlated. Proteins with high variability in both benign and prostate cancer tissue were enriched for immunity-related processes. DNA repair pathways exhibited a high degree of spatial variability in prostate tumor tissue, as well as cell cycle and nucleosome/chromatin assembly [49]. Goh et al. [73] used the same data and applied two network-based methods for the analysis: Quantitative Proteomics Signature Profiling (qPSP) and Paired Fuzzy Sub-Networks (PFSNet). In their work, network-based analysis outperformed protein-based feature selection and the cross-validation accuracy was higher. They also reported that with traditional statistical analysis, the extent of heterogeneity in such analyses may be exaggerated [73]. Shen et al. [74] also utilized LCM to study differences between patient-matched cancerous and benign epithelia and stroma. With no further clinical information provided about the samples from recurrent prostate cancer patients, this dataset provides mostly proof-of-principle of the methodological advancements in performing global proteomics, identifying >3200 proteins and metabolomics of key androgens from the same samples [74].

An increasing amount of evidence indicates that components in the prostate tumor microenvironment, especially cancer-associated fibroblasts (CAFs), play a causal role in prostate cancer. This happens both very early in the disease development, and during formation of therapy resistance and metastatic progression (reviewed recently in [75]). CAFs are reported to influence events such as extracellular matrix (ECM) deposition and remodeling, production of growth factors and cytokines, angiogenesis, and immune modulation. In vivo, CAFs can promote tumorigenesis of “initiated” prostate epithelial cells, while in vitro they can enhance tumorigenic potential and invasiveness of prostate cancer cells. Thus, CAF targeting may represent a therapeutic avenue. Nguyen et al. [76] performed quantitative proteomics of primary cell cultures of patient-matched prostate CAF and non-malignant prostate fibroblasts (NPF). Although the proteomic profiles of CAF and NPF were found to be highly similar, differential expression analysis of patient-matched pairs identified 363 DEPs. The CAFs were enriched for proteins with roles in the ECM and cell adhesion. On the other hand, the NPFs were enriched for proteins of metabolic pathways, the oxidation-reduction processes, and mitochondria. Network analysis with significantly increased DEPs in CAFs identified especially expression, regulation, and signaling of collagen. On the other hand, decreased DEPs in CAFs were associated with cellular metabolism, redox regulation, and mitochondrial function. The authors further identified 161 phosphopeptides with different abundance between CAF and NPF, however, most of them were not ECM-related. Through bioinformatic analysis and functional assays, they were able to identify overexpression of lysyl oxidase-like 2 (LOX2), an enzyme responsible for collagen linking, to be a potential therapeutic target on CAFs [76].

MS-based proteomics can also be performed on spatially intact tissue slides, avoiding both the bulk processing and the extraction of individual cells. Mass spectrometry imaging (MSI) can be used to measure biomolecules directly from a tissue section and to create multidimensional spatial expression maps to correlate molecular information to histopathological patterns in cancer [77,78]. This technique, although very potent in prostate cancer biomarker discovery [79], is currently not particularly high throughput and is mostly used to assess specific molecules or classes of molecules in a targeted fashion. For example, Kurreck et al. [80] have performed a systematic review for MSI metabolic studies in prostate cancer for diagnostic and prognostic set-ups. Recently, MSI has been used to identify, e.g., lipids distinguishing GS (4 + 3) from GS (3 + 4) tumors [81], to find zinc and its pathway metabolites citrate and aspartate correlated with each other and showing a significant reduction in cancer compared to non-cancer epithelium [82], and to find metabolic and lipid profiles differentiating cancer, non-cancer epithelium, and stroma [82]. In addition, identification of prostate cancer from needle biopsies utilizing a multivariate metabolomic classifier based on MSI has been suggested [83]. Only a few studies with MSI proteomics exist in prostate cancer. With this method, Cazares et al. [84] could resolve, on average, between 350 and 400 peaks in a discovery set of 11 prostate cancer-containing and 10 benign prostate tissue sections. Using 23 prostate cancer and 31 benign tissue samples as a validation set, they confirmed an expression profile that discriminates between prostate cancer (GS6–7) and normal tissue, further identifying mitogen-activated protein kinase/extracellular signal-regulated kinase kinase kinase 2 (MEKK2) as a marker to discriminate cancer from uninvolved tissue. Angel et al. [85] used MSI as a collagen-targeting proteomics approach to investigate zonal regulation of collagen-type proteins in prostate cancer prostatectomy samples. They found differences in collagen hydroxylation of proline, concluding that site-specific post-translational regulation of collagen structure may be involved in reactive stroma associated with prostate cancer progression [85].

### 2.2. Large-Scale Proteomics of Advanced Prostate Cancer

Quantitative, large-scale proteomics studies performed in advanced prostate cancer are listed in Table 1. These address locally advanced CRPC [56], lymph node metastases (LNM) [57,59], and distal metastases [58] compared to benign tissue and primary prostate tumors (PCa). In addition, phosphoproteomics (antibody-based extraction of pST- and pY-containing peptides) of metastatic prostate cancer compared to treatment-naïve prostate cancer has been published [55], as well as N-glycoproteomics comparing normal tissue and PCa samples to distal metastases [54]. Similar to what has been observed with PCa proteomics, there are numerous and various DEPs identified in the proteomic studies of advanced prostate cancer, but the identified functional protein groups and pathways show relatively more consistency. Further, several individual proteins or protein complexes have been validated to undergo alterations in advanced prostate cancer or identify sub-groups of advanced cases. The readers are directed to the original studies (Table 1) for lists of individual proteins. As the studies addressing proteomic changes in advanced prostate cancer are so far all unique, having been performed in different types of samples, the proteomic picture of the stepwise evolution of the advanced prostate cancer is not yet complete. However, the current studies have already provided us important insights into the proteomic alterations taking place during development of castration resistance and transition from primary to metastatic disease. Not surprisingly, heterogeneity of the proteome increases in advanced prostate cancer compared to primary disease. The proteomic profile of prostate cancer is significantly altered during the disease, when comparing primary prostate tumors to locally advanced CRPC [56]. Although protein expression profiles of localized tumors and bone metastases were found to significantly correlate, a higher overall heterogeneity in the proteomes of bone metastasis samples was observed compared to localized tumors and benign prostate tissues [58]. Metastatic tumors also have markedly distinct N-glycoproteomes from primary tumors [54].

The pathways most prominently altered at the stage of advanced prostate cancer compared to PCa are cell cycle- and DNA damage response-related. Despite reports of changes in individual proteins and the differences in cell cycle and proliferation reported in G3 vs. G4 epithelium [48], overt evidence of increased proliferation has not been observed with PCa proteomes [46,56]. Thus, at the large-scale proteome level, proliferative changes appear at the advanced stage. Increased alterations in cell cycle checkpoints have been observed when comparing CRPC to PCa [56] and, in distal metastases, higher levels of proteins involved in cell-cycle progression and DNA replication are detected [58]. In addition, cyclin-dependent kinase activities are enriched in metastatic prostate cancer based on phosphoproteomic analysis [55]. DNA damage responses undergo changes at the proteomic level in advanced prostate cancer, with DNA repair pathways (NHEJ, BER) altered in CRPC when compared to PCa [56], and DNA damage response proteins upregulated in distal metastases [58].

Some of the proteomic changes detected already in PCa are exacerbated in advanced disease. Examples include reduced expression of cell adhesion and carbohydrate metabolism, and increased levels of proteins functioning in RNA biogenesis and transport, lipid transport, and fatty acid oxidation [55,56,57,58]. Continuous alterations in metastatic prostate cancer compared to primary prostate tumors were detected in increased protein turnover and RNA processing: increased abundance of ribosomal and proteasomal proteins in recurrent LNM [57] and RNA processing in bone metastases [58]. Alterations in adhesion and cytoskeleton-related proteins (e.g., proteins of cytoskeleton, integrins, junctions, and ILK signaling) are detected in CRPC [56] and, for example, with lower protein levels in LNM than matched primaries [57]. ECM components and proteins involved with muscle contraction are often depleted in LNM [57] and locally advanced CRPC [56]. Similar to what has been discussed above concerning alterations in primary prostate tumors, this may at least partly reflect the decreased amounts of stromal components in the metastatic samples compared to normal or primary tumor tissue. Metabolism is significantly affected in CRPC compared to PCa with alterations in the TCA cycle, mitochondrial dysfunction, glycogen degradation, and ketogenesis pathways [56]. In Latonen et al.’s study, sequential changes in the TCA cycle were identified: the first change occurs during cancer development from benign tissue to PCa, while the second change occurs during formation of treatment resistance. These changes partly involve the same proteins, some with continuous and some with altered change directions, identifying distinct metabolic states for PCa and CRPC [56]. In distal metastasis, especially the fatty acid ß-oxidation pathway is altered [58]. Interestingly, the metabolic pathway alterations so evident with basic proteomics were not identified by phosphoproteomics [55]. This is likely due to the fact that metabolic enzymes, in general, undergo less post-translational regulation than some other types of proteins, such as signaling molecules. Gene set enrichment analysis of phosphoproteomes did, however, support over-representation of mRNA splicing, RNA processing, and DNA replication pathways, as well as loss of cell adhesion and motility-related pathways, such as integrin signaling, focal adhesion, and axon guidance, in metastatic CRPC compared to treatment-naïve PCa [55]. Further, activities of transcription factors AR, E2F family, and MYC/Max are upregulated in metastatic CRPC based on phosphoproteomics. Enrichment of several kinases in metastatic CRPC is also indicated by phosphoproteomics, including cyclin-dependent kinases (CDK2/CDK3), casein kinase 2 (CSNK2A1), and b-adrenergic receptor kinases (ADRBK1/ADRBK2) [55].

The major differences between localized prostate cancer and distal metastases have been underlined by the fact that, according to the proteomic profiles, the treatment-naïve metastases and metastases which have been collected shortly after the beginning of a castration protocol were found to cluster with CRPC metastases and not with primary tumors [58]. Interestingly, the general features of proteomic changes (including increased expression of proteins involved in proliferation, RNA biogenesis and transport, lipid transport, and fatty acid oxidation, as well as proteins with reduced expression in cell adhesion and carbohydrate metabolism) were observed regardless of whether the patients had received ADT [58], suggesting that these changes support aggressive features required from transition from localized cancer to metastatic spreading.

Flores-Morales et al. [86] showed, by analyzing 17 prostate cancer patient-derived xenograft (PDX) tumors, that response to castration therapy involves a large degree of proteomic variability. As expected, PDX tumors from castrated mice showed signs of reduced androgen signaling through lowered expression of products of several AR target genes. Decreased expression of proteins involved in fatty acid synthesis was also detected, as well as decreased expression of several proteins involved in rRNA processing and DNA metabolism. Certain proapoptotic proteins were elevated in their expression, along with several proteins related to axon guidance—even without signs of NE marker upregulation [86].

### 2.3. Integrative Studies Comparing Genomic, Transcriptomic, and Proteomic Alterations in Clinical Prostate Cancer

Integrative studies combining genomic and transcriptomic analyses to proteomics from the same clinical prostate tumor samples are valuable in assessing outcomes and effects of genetic lesions and transcriptomic aberrations of protein-coding genes at their functional level. The first such integrative study published compared alterations between BPH samples, untreated primary tumors (PCa), and locally recurrent CRPC [56]. The study thus uncovered alterations occurring both during the development of PCa as well as during the formation of castration resistance [56]. The results showed that, especially in the treatment-resistant form, prostate cancer gene copy number, DNA methylation, and RNA expression levels do not reliably predict proteomic changes. Another integrative large-scale proteomics study, which was performed on a cohort of localized, clinically homogeneous, treatment-naïve PCa samples [52], similarly showed that in comparison with genome, methylome, and transcriptome, the proteomic features are poorly correlated. Additionally, in distal metastases, integration of proteomic and transcriptomic profiles revealed only a weak correlation between mRNA and protein expression levels in general [58].

The low extent of correlation between genomic aberrations and the proteome in prostate cancer is remarkable [52,56]. Only 2% of proteins have their abundance associated with gene copy numbers in PCa [52], a disease state that is considered driven by copy number aberrations more than single nucleotide variants [87]. The mutational burden and protein abundance profiles were not significantly associated in PCa [52]. Furthermore, the altered gene copy numbers and the global methylation changes occurring in prostate tumors were not as detectable through the proteome as through the global RNA expression in PCa. This was even more pronounced in CRPC that contains significantly higher level of genomic aberrations [56]. Interestingly, it was shown that deletion of PTEN affects the abundance of only 2.7% of proteins, even though it affects around half of the studied genes at the mRNA level [52]. The five subtypes of PCa identified by Sinha et al. [52] based on the proteome were largely independent of mutational burden (genomic rearrangements and somatic nucleotide variants) of the tumors, and the genomic and proteomic subtypes were also largely independent. Collectively, these results indicate that a large proportion of aberrations and even regulatory events in the genome do not significantly affect protein levels in prostate tumors.

One of the key messages of the integrative analysis of proteomics and transcriptomics has been that RNA expression is, in general, a poor predictor of protein expression. A poor correlation between expression changes at the transcriptomic and proteomic levels has been detected in several types of cancer [32,33,34], and also holds true for prostate cancer [52,56,58]. Globally, mRNA and protein abundances are only weakly correlated in PCa [52,56], and this correlation is further decreased as the disease progresses to CRPC [56]. In both primary and advanced stages of the disease, there were DEPs found that are not associated with corresponding changes at the mRNA level [56]. In distal metastases, global correlation between mRNA and protein expression levels is also poor [58]. There are many proteins whose expression patterns adhere well to the trends visible at their mRNA expression, but many do not, and even inverse patterns are detected. In PCa, mRNA abundance explains only around 10% of protein abundance variability [52]. This underlines the importance of examining the proteome instead of relying on mRNA expression as a proxy of protein levels. Interestingly, the most abundant proteins in the PCa proteomes were better correlated with their mRNAs than the least abundant proteins [52]. This likely reflects differences in the roles and regulation of these protein groups, with the high-abundance proteins often representing core function and structural proteins, such as cytoskeletal and attachment proteins, ribosomal proteins, proteins in membrane-bound organelles, and extracellular proteins. These are more easily detected by MS approaches. On the other hand, many highly and transiently regulated, signal-responsive proteins, such as signal transductors and highly specific post-translational protein-modifying enzymes, are generally lower in abundance and less likely to be detected. An indication of this may be the notion that proteins with coding transcripts expressed but no protein detected had an over-representation of nuclear proteins [52].

Quantitative, large-scale proteomic data have revealed novel pathway alterations that have not been visible through RNA expression data [56,58]. This was especially evident in the advanced form of the disease: in CRPC, the alterations in cell metabolism, DNA metabolism and repair, and various signaling pathways were better spotted in analyses based on proteomics than transcriptomics [56]. In fact, less than a third of the pathways found altered were common between analyses performed based on either RNA or protein abundances [56]. Furthermore, several pathways were identified to be altered based on mRNA abundance, which were not identified based on protein abundance [56]. This underlines the importance of caution in interpreting the effects of genomic aberrations by transcriptomic data alone, especially as a proxy of functional activities. It is noteworthy that genes involved with mRNA translation, oxidative phosphorylation, and components of ribosomes and proteasome had especially low degrees of mRNA–protein correlation [58]. An integrative study with comparisons of genomic, transcriptomic, and phosphoproteomic data from metastatic castration-resistant samples [55] revealed functionally mutated genes and master transcription factors that are differentially expressed. Furthermore, analysis of differentially activated kinases indicated that there are six major signaling pathways with several key residues phosphorylated: PI3K-AKT-mTOR, stemness (including TGFb, WNT, NOTCH, and MYC pathways), nuclear receptor, cell cycle, DNA repair, and migration and invasion pathways [55].

The integrative proteogenomic studies have brought about interesting findings concerning the ETS gene fusions. As these are the most frequently occurring somatic aberrations in prostate cancer (for reviews, see [88,89]), they are of significant interest. The ETS fusions were found to be significantly associated with alterations in protein abundances [52]. Interestingly, the proteins that are most affected by the presence of ETS fusions showed a good correlation between their protein and mRNA levels [52]. For example, genes associated with intra- and extra-cellular vesicles were enriched in ETS fusion-positive tumors both at mRNA and protein levels. Many genes associated with ETS fusion status were enriched at all studied molecular levels, including mRNA and protein abundance and DNA methylation. These were connected to carboxylic acid metabolism, confirming links between ERG and lipid metabolism. However, there were many genes found that associated with ETS fusion status only at either their mRNA or protein level. Interestingly, ETS fusion-containing tumors exhibited enrichment at the mRNA level on migration, actin binding, and phospholipid binding activities, while at the protein level, enrichment was shown for lysosomal genes, showing that ETS-associated transcriptome is differentiated from ETS-associated proteome [52].

To date, no large-scale proteomic studies exist assessing proteomics or proteogenomics of clinical patient samples of NEPC. Patient-derived tumor xenografts have been studied with a cohort containing a PDX developing to NE type upon castration resistance in mice, and two PDX tumors originating from the same patient with NE-type metastasis [86]. Comparing the proteomes of these NE tumors to AR-dependent adenocarcinoma tumors, >800 DEPs were identified. NEPCs had increased expression of proteins associated with neuronal differentiation, mitotic cell cycle, cell division, and chromatin remodeling, in line with the highly proliferative phenotype of NEPC. Significant upregulation was found for proteins involved in several DNA repair pathways (HR, NER, MMR) and nucleotide metabolism. Lower levels of mitochondrial proteins (catabolism of fatty acids, amino acids, and the TCA cycle), as well as lysosomal and oxidative stress response proteins, were detected. Further, downregulation of proteins within several cytosolic compartments, such as ER, Golgi, and the lysosome, was detected. Reduction of these may be associated with the reduced cytosol volume, characteristic of small-cell carcinomas. It is noteworthy that, also in this study, the metabolic pathways were obvious through proteomic analysis and not through transcriptomics. The data suggested that NEPCs rely more on glycolysis to fulfil their energetic needs, and that NEPC cells restrict their cellular activities to cell division [86].

For the non-coding genome, integrative proteogenomic analyses can reveal complex associations not visible through genetic and transcriptomic data alone. For example, Latonen et al. [56] identified several novel miRNA–target gene interactions that affect the protein end-product, with no significant changes detected at the mRNA level. This both provided more miRNA targets to further assess for potential clinical applications, as well as showed that miRNA target regulation based on transcript level associations underestimates the extent of regulatory events taking place by miRNAs in cancer [56].

## 3. Large-Scale Proteomes of Prostate Cancer Models Provide Mechanistic Insights

While proteomes of clinical cancer samples are essential, they currently provide only snapshots of the clinical situation at the time of sampling and are so far unable to provide dynamic information. Causalities between events can also be easier to assess in prostate cancer models, as many events may remain masked in the heterogeneity of proteomes available from clinical datasets. Significant amounts of earlier work based on 2-DE/MS approaches have been performed to identify DEPs in different types and sublines of prostate cancer cells, upon effects of androgens and anti-androgens, and for identification of factors distinguishing androgen-dependent from -independent lines. Here, we focus on the recent, large-scale studies with broader insights on pathway alterations taking place in prostate cancer (Table 2). We were unable to summarize all studies performed in cell lines with different drug treatments, and therefore focused on studies of cancer mechanistic insights.

Cell lines may have very distinct proteomic and phosphoproteomic profiles compared to each other and to clinical samples, and cell lines are reported to have distinct phosphoproteomic profiles from primary and metastatic CRPC tissues [55,60]. Several studies have profiled the large-scale proteomes of prostate cancer cell lines, especially for PC-3 and LNCaP [96,98], and identified numerous proteins with different expression levels. Although the cell lines have differences in terms of their AR status and androgen-dependence (for example, PC-3 is androgen-independent (AI) and LNCaP is androgen-dependent (AD)), conclusions based on differences in DEPs between these cell lines in terms of androgen dependence should be validated by other assays.

Katsogiannou et al. [95] analyzed the proteomes and phosphoproteomes of four prostate cell lines with different cancerous and hormonal status (PNT1A is an AR-negative SV40-immortalized normal prostate cell line, LNCaP is an AR+ cancer cell line, and DU145 and PC-3 are AR-negative cancer cell lines) by SILAC-based quantitative proteomics. The work quantified more than 1000 proteins and 500 phosphosites. Significant variation in protein expression was detected between the cell lines, and proteins with the most differential patterns were associated with stress responses, actin cytoskeleton, and RNA binding. In addition, significant metabolic differences, associated especially with the TCA cycle, were detected. Molecular network analysis highlighted migration, invasion, RNA splicing, DNA damage repair response, and transcription regulation as differentially expressed pathways. Proteins common between the cancer cell lines compared to PNT1A were associated with protein secretion, protein stability, chaperone-mediated protein folding, and DNA metabolism. Furthermore, the authors identified over 270 DEPs between castration-resistant and castration-sensitive cells, functioning in a plethora of cellular pathways, such as cell–cell adhesion, external communication, energy metabolism, and protein maturation processes. Liynage et al. [100] studied the response of the AD LNCaP cell line and its AI derivative C4-2B to anti-androgens bicalutamide and enzalutamide. With SWATH-MS, they identified approximately 2700 proteins in each cell line, with distinct proteomic profiles. Their analysis indicated MYC and PSF/SFPQ as upstream regulators activating in the anti-androgen treatments, and regulation of energy metabolism, transcription, translation, cell communication, and tumor growth and proliferation-promoting cell signaling as main events in response to anti-androgens. They also reported, for example, suppressed homologous recombination, activated PARP DNA repair system, and marked changes in RNA metabolism upon anti-androgen treatments [100].

Höti et al. [92] studied androgen resistance in LNCaP cells with the iTRAQ method and reported >1800 proteins significantly altered between the castration-resistant subline LNCaP-95 compared to parental, androgen-sensitive LNCaP cells. In LNCaP-95 compared to LNCaP cells, overexpression of proteasome proteins and amplification of the PI3K/AKT pathway were reported. On the other hand, the mitochondrial oxidation and phosphorylation pathways were downregulated. Interestingly, in the castration-resistant line, induction of the cytoplasmic endoribonuclease microRNA regulator Dicer was found, indicating alterations in miRNA regulation in these cells. Singh and Sharma [98] analyzed protein expression phenotypes of prostate cancer cell lines PC-3 and LNCaP by SWATH quantitative MS upon TGF-b-induced epithelial to mesenchymal transition (EMT). In the AD LNCaP cell line, 2 proteins were significantly upregulated, and 126 were downregulated. In the AI PC-3 cell line, 69 proteins were significantly upregulated and 277 were downregulated—26 of these proteins were common between the cell lines [98].

Kwon et al. [96] assessed proteomic effects of increased cell line aggressiveness by comparing LNCaP and PC-3 to their more metastatic sub-lines LNCaP-LN3 and PC-3M, quantifying >3400 proteins. The DEPs in these AI and AD models were mostly different and found to have opposite expression patterns. In LNCaP-LN3 compared to LNCaP, proteins with increased expression were associated with, e.g., ER and protein disulfide isomerase activities, as downregulated ones were mostly extracellular proteins. In PC-3M compared to PC-3, proteins with increased expression were associated with, e.g., cell proliferation and extracellular events, while decreased expression was associated with focal adhesion, integrins, and ECM–receptor interactions. The modest and inconsistent alterations in the derived cell lines, also detected earlier [101], may be explained by one cell line being AI and the other AD, but also considering that both LNCaP and PC-3 cell lines are already derived from metastases. Kwon et al. [96] further compared these cell lines to identify mutated peptides by tandem mass tag-based quantitative proteomics: 70 mutant peptides from 66 proteins were identified. They confirmed some of these in prostate cancer tissues by targeted quantitative MS and were able to confirm 7 having differential expression in tumors compared to normal tissues. This is a powerful approach to confirm mutation effects reaching the level of proteins and, thus, confirming biological significance and/or potential for targeting. Miao et al. [97] used SILAC combined with parallel reaction monitoring-based targeted proteomics to assess kinome associated with metastasis in prostate cancer cells. They used PC-3 cells compared to a more metastatic subline PC-3MLN4 and quantitated 276 kinases in this pair, 71 up- and 62 down-regulated. Of these, MERTK and SRC have previously been known to promote prostate cancer bone metastasis, and the authors further assessed the metastasis-associated functions of MAP4K4. Additionally, Zhang et al. [93] compared PC-3-derived prostate cancer cell lines with different metastatic ability. They used iTRAQ-based proteomics to explore DEPs between highly metastatic PC-3M-1E8 and poorly metastatic PC-3M-2B4 cell lines. A proteome of >6000 proteins revealed 58 DEPs and regulation of glutathione metabolism, actin cytoskeleton, hematopoietic cell lineage pathways, and viral carcinogenesis. Enrichment was detected for cell migration, cytoskeleton organization, and epithelial to mesenchymal transition pathways in the highly metastatic potential of PC-3M-1E8 cells.

Jiang et al. [91] assessed LNCaP xenografts for phosphoproteome by studying tumors grown in intact or castrated mice with SILAC-based quantitation of phosphorylation sites, altering based on the host hormonal status: 800 phosphopeptides were quantitated and 98 were differentially expressed, with 44 increased and 54 decreased. Interestingly, ACC1, a rate-limiting enzyme of fatty acid synthesis, was the most prominently altered protein observed. Proteins involved with cytoskeletal organization, morphogenesis, and intracellular transport processes were enriched in the increasingly phosphorylated group. Diminished phosphorylation was observed amongst proteins of nucleic acid metabolism, including nucleosome organization, transcription, and RNA processing. The study also identified YAP1 and PAK2 as targets relevant for pharmacological inhibition of androgen-independent tumor growth. Zhang et al. [94] performed unlabeled quantitative proteomics of TRAMP mice, a prostate cancer model induced by expression of SV-40 Large T antigen. Comparing proteomes of full prostates of four wild-type (wt) mice to those of four TRAMP mice at 18 weeks, >2300 proteins were identified and a reference mouse prostate proteome of >900 proteins was produced. Sixty-one DEPs and twelve proteins identified exclusively in either group were analyzed to identify UPR, actin cytoskeleton signaling, and antigen-presenting pathways, as modulated. The authors speculate that the activation of antigen-presenting signaling may be related to immune response to the T antigen. More specifically, the authors identified the PDGF-B network, with six intermediate regulators (RAF-1, MAPK, MAPK3, MAPK1, AKT1, and PI3K) to be upregulated in the TRAMP tumorous prostates [94].

Zhang et al. [99] compared Pten−/− and wt whole prostates of 4 + 4 mice between 12 and 15 weeks (i.e., when tumors were palpable) with the 8-plex iTRAQ approach. Identification of 711 proteins and further analysis of around 100 DEPs revealed significant enrichment of inflammatory and immune pathways, with a predicted significant upstream role for NF-kB. Other modulated pathways included, for example, glutathione conjugative metabolism, proteases related to invasiveness, chromatin modulation, and ER stress responses. Interestingly, analysis of transcriptomic alterations in the same mouse model also strongly indicated NF-kB-driven inflammatory and immune responses. On the other hand, activation of the AKT pathway was prominent in the transcriptomic analysis but was not revealed by proteomics. Involvement of p53 as an upstream regulator was indicated by both transcriptomic and proteomic data, although with the latter only weakly [99]. The authors also reported that most of the downregulated pathways in the *Pten* KO mouse prostates were also decreased in the mutant TRAMP prostates detected by Zhang et al. [94]. The most suppressed proteins in the *Pten*−/− proteomics reflected diminished prostate epithelial differentiation, including several AR targets such as Pbsn and Nkx3.1. These proteins have also been reported to be downregulated in TRAMP and hiMYC prostate cancer mouse models, linking the different models through possible carcinogenesis-induced epithelial de-differentiation [99]. In the search for candidate biomarkers from prostate tissue and sera of wt and *Pten*−/− mice, Cima et al. [90] identified 775 N-linked glycoproteins with label-free quantitative proteomics. They identified >150 glycoproteins specifically in the *Pten* KO prostates. Then, they applied targeted proteomics to the sera of prostate cancer patients and controls to detect and quantify human orthologs. With identification of 39 candidate biomarkers, they validated the approach to use mouse prostate cancer models in the search of proteomic biomarkers.

Tan et al. [60] assessed the effects of ERG on proteomes of VCaP cells which harbor the *TMPRSS2-ERG* fusion gene. Proteins were isolated from non-targeting siRNA- and ERG siRNA-treated VCaP. They detected 562 proteins specifically in the control siRNA-transfected cells, 59 proteins specifically in ERG-silenced cells, and 1569 DEPs that were common between the sample types. Interestingly, comparison of all these 2190 proteins against the transcriptome of siRNA-treated VCaP cells of 1052 distinct genes revealed 250 genes and proteins with concordant responses to ERG expression. This represented 24% of the ERG responsive genes and 11% of the ERG responsive proteins. The authors also compared the ERG siRNA proteome in VCaP cells to their ERG-associated proteome from clinical PCa samples. These proteomes showed an overlap of 489 proteins, of which 330 DEPs showed concordance in their response to up- or down-regulation of ERG protein levels, accounting for 15% of VCaP ERG proteome and 28% of LCM isolated ERG+ and ERG− tumor proteomes. ERG-responsive proteome networks indicated effects of ERG on protein biosynthesis, protein trafficking, chaperone and redox functions, cell survival and apoptosis, cell cycle control, DNA replication, cell polarity, cell migration, and AR signaling. In both ERG-positive vs. -negative tumors and in non-targeting siRNA- vs. ERG siRNA-treated VCaP cells, Proliferating Cell Nuclear Antigen (PCNA) was upregulated, and PSA was downregulated. The 330 DEPs common between the prostate tumors and the cell culture model indicated enrichment of cytoskeletal and actin reorganization pathways (CDC42-RAC1 pathway, PAK pathway, the actin filaments Y-branching pathways, the proteasome, and the ER-associated degradation (ERAD) pathway). The work further showed that knock-down of ERG affects the epidermal growth factor receptor (EGFR) signaling pathway and induces the expression of prostate differentiation markers that are associated with the secretory function of the organ [60].

## 4. Large-Scale Proteomics in Protein Dynamics: Functional Interactomes and Subcellular Localization Patterns in Prostate Cancer

While large-scale MS analyses from tumor tissue samples and cells provide us with valuable snapshots of the proteome of prostate cancer at a given time, they do not provide information on molecular interactions between the proteins expressed at a given time. As proteins do not work in solitude but as parts of interconnected networks based on variable localizations and interactions, assessment of merely presence does not suffice for proving and understanding of the functional connections. Thus, to understand molecular driving events in prostate cancer, it is important to assess the functional sub-proteomes, such as the interactomes of key cancer-driving factors. In addition to methods combining affinity purification and immunoprecipitation to MS, proximity labeling methods to identify and assess dynamic interactomes of individual proteins in mammalian cells have been recently developed [102]. These methods, such as BioID [103], allow enhanced mapping and visualization of protein interaction networks. In the immuno-purification-based assays, it is likely that not all identified proteins are direct binding partners but can interact through intermediate proteins (or other molecules such as RNA). Proximity labeling assays are limited in proteins interacting within short distances. However, none of the large-scale interactome assays themselves offer solid proof of direct interaction, and verification methods need to be applied.

**Figure 2:**
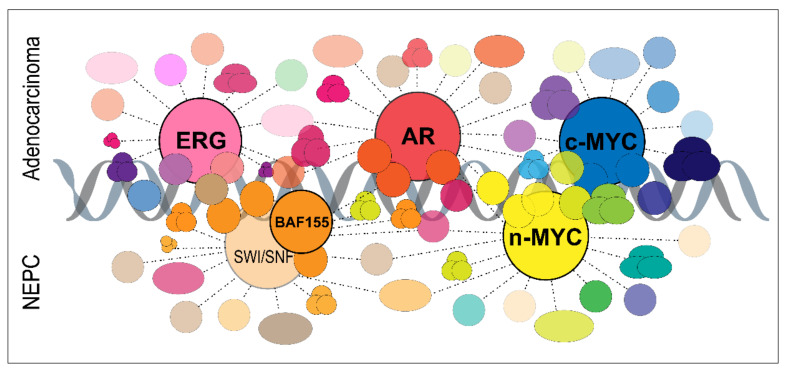
Schematic illustration of prostate cancer-relevant protein interactomes assessed by proteomics. Mass spectrometry-based interaction proteomics for key prostate cancer transcription factors have revealed disease-relevant protein interactions for AR, ERG, c-MYC, n-MYC, and the BAF155 component of the SWI/SNF complex. While many of these transcription factors partly interact with the same pool of co-regulators, chromatin modifiers, and other transcriptional regulators, specific interactions have also been indicated. AR, ERG, and c-MYC interactions likely have more significance in prostate adenocarcinoma cells, while the SWI/SNF complex and n-MYC are especially important in neuroendocrine prostate cancer.

The interactomes published so far with the above methods are in the order of dozens or hundreds of interacting proteins identified. Significant differences exist in how strict analysis has been applied to the interaction confidence in the interpretation of potential interacting proteins. The interactome research relevant for prostate cancer is so far concentrated on AR and certain other transcription factors (Figure 2), and is thus closely connected to events on chromatin (reviewed in [104]). In addition to target binding site sequences on DNA, protein–protein interactions play a large role in targeting transcription factors to sites on chromatin. The interactomes obtained so far for AR and MYC support their key roles as transcription factors. However, other interactions also exist and may be significant for cancer-relevant roles, e.g., through post-translational modifications. Dynamic interactions also take place elsewhere than on chromatin, which is an important topic for future studies. Many proteins are regulated post-translationally through cellular localization, such as AR for translocation between the nucleus and cytoplasm when activated, which is why combining proteomic information with localization patterns is of importance.

### 4.1. Interactomes of AR wt and Mutants

AR is known to interact with several hundreds of proteins based on decades of research, often with individually assessed interactions or low-throughput interactome analyses. Many AR-interacting proteins function as co-regulators of AR-mediated transcription. Novel interactors are, however, still surfacing continuously, and a significant number of interactions also exist in the cytoplasm. Understanding how this level of regulation of AR functions is affected by AR activity, by surrounding cellular context, and how it becomes aberrated during prostate cancer development and progression, are questions that can be assessed by utilizing protein interactomics.

Mutations of the AR ligand-binding domain often occur in response to ADT. Mutations of T877 have been reported to occur in androgen-independent or castration-resistant tumors. The Thr877Ala (T877A) mutant AR, present in the LNCaP cell line, has gain-of-function properties: it shows promiscuous binding to several classes of steroids, which can induce transactivation, and it can be hyperactivated by the normal ligands. Zaman et al. [105] utilized eight different ligands (testosterone, dihydrotestosterone, R1881, mibolerone, estradiol, progesterone, dexamethasone, and cyproterone acetate) with LNCaP and studied the effect of the promiscuous binding to the mutant AR interactome by traditional immuno-purification of AR using an N-terminal antibody, followed by label-free quantitative proteomics. Surprisingly, proteomic interaction profiles of androgen ligands do not cluster together, but progesterone and dexamethasone AR complexes have proteomic profiles that resemble those of R1881 and mibolerone, respectively. Moreover, the protein interaction pattern for estradiol-stimulated AR complexes was most similar with the AR interactome upon DHT response. These unexpected patterns were supported by gene expression analysis in the same study, showing that the stimulation profiles induced by different androgens do not cluster together. The synthetic androgens R1881 and mibolerone do not share the same AR-stimulated transactivation profiles as testosterone and DHT. The major ontological functions based on protein interaction data from all the ligand stimulation conditions were RNA polymerase II transcription, RNA metabolism, especially with RNA splicing, protein biosynthesis, including translation, the ubiquitin-proteasome pathways, and DNA repair. In contrast, the gene expression patterns depending on specific ligands included steroid/sterol biosynthesis, DNA replication, and apoptosis. Thus, despite that the AR is a transcription factor, these proteome profiles suggest that it also participates in functions beyond gene activity regulation [105].

Paliouras et al. [106] combined His-tagged affinity purification and immuno-purification to assess interactions of wt AR and two somatic gain-of-function AR prostatic mutants (T877A-AR and 0CAG-AR isoforms). Identified interactomes for each AR isoform in cells with or without androgen stimulation by mibolerone in COS-1 cells were comprised of 200–400 proteins in each condition. Comparative analysis identified sub-network cluster profiles for AR interaction that correlated with prostate cancer progression and outcome. Specifically, they identified two AR interaction clusters, containing 21 and 30 proteins respectively, that showed an unfavorable outcome of recurrent cancers based on PSA, Gleason score, and combined PSA/Gleason score. Hsiao et al. [107] performed label-free MS analysis of AR interactome based on streptavidin-binding peptide-tagged wt AR. The analysis was performed from the cytosolic fraction of LNCaP cells, either androgen-deprived or stimulated by R1881. The analysis resulted in >3100 proteins, of which roughly a fourth overlapped between androgen-deprived and -stimulated conditions. Considering how AR translocates to the nucleus upon stimulation, the result may not be surprising. The six top-ranked pathways differentially regulated by the two stimulation conditions included AR signaling, glycolysis and gluconeogenesis, mRNA processing, translation, and the ubiquitin-proteasome system.

To date, two proximity-mapped AR interactomes based on the BioID method have been published. Lempiäinen et al. [108] studied agonist- and antagonist-specific protein interactions of AR, at the same time as that of the related steroid receptor glucocorticoid receptor (GR). They identified high-confidence unbiased interactomes of AR either activated by DHT or inhibited by enzalutamide. While enzalutamide significantly decreased high-confidence interactions of AR, the interactome of DHT-activated AR was enriched with known cofactors, corepressors, components of the BAF (SWI/SNF) chromatin remodeling complex, lysine methyltransferases, and demethylases. While this study used human embryonic kidney (HEK) 293 cells [108], which do not endogenously express detectable levels of AR, another study applied BioID in androgen-dependent LAPC4 cells that express wt AR [109]. The difference may affect the binding partners present in the cells. Velot et al. [109] identified 31 AR-associated proteins in non-stimulated cells. With androgen stimulation by DHT, the AR signaling network increased to 182 and 200 proteins, upon 24 or 72 h respectively, for a total of 267 AR-associated proteins. Of these proteins, >200 were not previously reported in the queried databases, and many of them were involved in DNA metabolism, RNA processing, and RNA polymerase II-dependent transcription. Both published AR proximity networks indicated a significant number of known and new AR-associated proteins that function in the regulation of gene expression. These emphasize associations between AR and the transcriptional and chromatin remodeling complexes. Venot et al. further identified Krüppel-like factor 4 (KLF4) as a new AR interaction partner. AR and KLF4 colocalize genome-wide on >4000 genes, including KLK3, for which KLF4 acts as a repressor. Lempiäinen et al. went on to report BCOR as a corepressor of a subset of AR targets [110].

The chromatin-associated AR interactome has been studied specifically by many researchers with the rapid immunoprecipitation MS of endogenous proteins (RIME) method, and the results support the current view of AR interacting with a multitude of transcription co-factors, chromatin modifiers, and other transcription factors. Paltoglou et al. [111] utilized RIME in R1-AD1 and R1-D567 cells, originating from an androgen-responsive monoclonal subline of the CWR-R1 prostate cancer cell line. They identified interactors of wt AR and the constitutively active ARv567es variant, for which 54 and 75 interactors were reported, respectively. They further characterized the transcription factor Grainyhead-like 2 (GRHL2) as an oncogenic enhancer of androgen signaling, a co-regulator of AR, and a suppressor of metastasis. Stelloo et al. [112] performed RIME in LNCaP cells to identify proteins in the transcriptional complex of endogenous AR activated by synthetic androgen R1881. The 66 AR interacting proteins identified included known (e.g., ARID1a, HOXB13, HSP90, FOXA1, PARP1) and novel (e.g., TLE3 and TRIM28) AR interactors. They further also validated some of these in LAPC4 cells and in patient-derived xenografts of prostate cancer. A subset of AR interactors was further investigated with chromatin immunoprecipitation assays. Interestingly, three major subgroups of genomic subcomplexes of AR interaction partners were identified, where FOXA1 and HOXB13, known transcription factors interacting with AR and affecting the AR cistrome in prostate cancer, dictate selective gain of function for AR action [104]. Launonen et al. [113] utilized chromatin immunoprecipitation coupled with selective isolation of chromatin-associated proteins (ChIP-SICAP) to study the protein interaction network of chromatin-bound endogenous AR in VCaP cells in response to R1881. Eighty-seven R1881-induced AR-interacting proteins were recognized. In addition to corroborating AR interaction with many known co-regulators, they identified several previously unidentified AR interactions. They further studied how interactions with SMARCA4 and SIM2 affected AR target gene expression and cellular functions therein, finding that while the former affected cell morphogenetic changes and EMT, the latter influenced cellular responses to external and steroid hormone stimuli [113].

### 4.2. Other Prostate Cancer-Relevant Protein Interactomes

The MYC transcription factor family is important for many cancers in addition to prostate cancer, which is why their interactomes have been of relevance to many. Agraval et al. [114] performed tandem affinity purification (TAP) screening for c-Myc interactions in human fibroblasts transformed with telomerase and SV40 large and small T antigens (LF1/TERT/LT/ST model) and identified over 400 c-Myc-interacting proteins with functions in transcription machinery, DNA replication, and RNA processing pathways. The BioID approach utilizing the 293 T-REx cell model both in vitro as cell cultures and in vivo as xenografts by Dingar et al. [115] yielded >100 high-confidence MYC-interacting proteins, identifying DNA repair and replication factors, transcriptional co-regulators, general transcription and elongation factors, and components of the STAGA/KAT5 and SWI/SNF chromatin remodeling complexes. Later, Kalkat et al. [116] identified >300 high-confidence protein interactors of c-Myc with the BioID approach, also assessing the significance of different MYC homology box regions in the interactions. While the dynamic interactome of c-Myc remains unstudied, specifically in prostate cancer cells, the interactome of N-MYC has been studied in the LNCaP cell line. N-Myc is not normally expressed in the lineage of prostate epithelial cells but is overexpressed in a subset of CRPC adenocarcinomas and the majority of NEPC. Especially, a subset of CRPCs that become independent of AR signaling and develop neuroendocrine features through lineage plasticity [117] are associated with poor prognosis and aggressive disease and are partly driven by aberrantly expressed N-Myc. Berger et al. [118] performed integrative analysis of the transcriptome, cistrome, and interactome of N-Myc in prostate cancer. They used in vivo, in vitro, and ex vivo models, including patient-derived organoids, and identified that a lineage switch towards a neural identity is linked to epigenetic reprogramming. They also found that N-Myc interacts with several known AR cofactors to alter DNA binding. To identify other proteins that could regulate N-Myc binding, they utilized RIME in LNCaP-N-Myc cells in the absence or presence of androgens. Most interactions were shared between these conditions and included well-known N-Myc-interacting proteins (e.g., MAX and TRRAP), proteins associated with heterochromatin (e.g., chromobox homologs CBX1, CBX3, and CBX5), as well as HOXB13. While differences in binding affinity between the conditions were detected, no interactions specific to either condition were observed. These data combined with the cistrome data in the same study indicate that the interactions of N-Myc are not very affected by the presence or absence of androgens. However, cofactors can direct N-Myc to bind sites on chromatin that are accessible androgen-dependently. This can occur, for example, through altered chromatin accessibility or competition at the binding sites.

The chromatin modeling SWI/SNF complex has a key role in NEPC development [117]. Cyrta et al. [119] performed co-immunoprecipitation against the core SWI/SNF subunit BAF155 (SMARCC1) at low stringency, followed by MS in the CRPC-NE cell line NCI-H660 and in LNCaP cells overexpressing AR. Proteins that immunoprecipitated with BAF155 in NCI-H660, but not in AR-LNCaP, included BAF53B (ACTL6B) and BAF45B (DPF1) subunits, as well as several neural differentiation-specific factors, such as the growth factor VGF, the transcription factor NKX2.1 (TTF-1), and the microtubule-associated factor MAP2. In addition, several members of the NuRD chromatin remodeling complex were found to interact with BAF155. SWI/SNF was found to interact with proteins of chromatin regulation and DNA repair in CRPC-NE cells. On the other hand, in adenocarcinoma cells, but not in CRPC-NE, proteins that immunoprecipitated with BAF155 included HOXB13. Further, most of the genes coding these factors were found to be differentially expressed between adenocarcinoma and CRPC-NE cell lines and organoids [119].

Of the other key players in prostate cancer, PTEN interactomes assessed specifically in prostate cancer are lacking. However, the general interactome view of PTEN has been recently reviewed [120]. Many other proteins have been studied for their protein interaction partners in prostate cancer cells by immunoprecipitation of tag-based affinity purification set-ups combined to proteomics, identifying the strongest interactors, and their number is beyond reach to review here. The novel methods assessing dynamic interactomes have not been applied to many proteins yet. The interactome of the prostate-specific protein Anoctamin 7 (ANO7) was studied by Kaikkonen et al. [121]. As elevated expression of ANO7 is associated with poor survival in prostate cancer patients, they aimed to identify interactors of ANO7. The BioID method utilized in transiently transfected LNCaP cells identified 64 potentially ANO7-interacting proteins. Cellular vesicle was found as one of the most enriched cellular components, and co-localization was shown for heat shock protein family A member 1A (HSPA1A), staphylococcal nuclease and tudor domain containing 1 (SND1), adaptor-related protein complex 2 subunit beta 1 (AP2B1), and coatomer protein complex subunit gamma 2 (COPG2). Of specific interest for prostate cancer molecular mechanisms is the study by Fu et al. [122], who developed immunoprecipitation-MS assays to measure the endogenous TMPRSS2-ERG fusion protein in VCaP prostate cancer cells, even in low abundance and with its isoforms and interactome. Immunoprecipitation-shotgun MS (IP-MS) and immunoprecipitation-selected reaction monitoring (IP-SRM) assays were used to reveal four distinct isoforms of TMPRSS2-ERG fusion protein. ERG interactome using C-terminal antibodies identified 29 proteins, including AR and its transcriptional co-regulator complexes, BRG1- and BRM-associated canonical SWI/SNF complexes, and other transcriptional regulators. Such specific sub-interactomes will surely help to understand the fine-tuned biological transitions induced in prostate cancer, especially driving adaptation to castrate conditions, by alternative transcripts and mutated gene products.

## 5. Conclusions

Prostate cancer is the most frequently diagnosed malignancy in men and is associated with significant morbidity and mortality. The main goals in prostate cancer research are to discover new markers and molecular patterns to accurately stratify prostate cancer patients with indolent tumors from those with aggressive disease, as well as to identify novel mechanisms for developing better treatments. After the sequencing era, our interest is turning to the outputs of the already relatively well-known genomic and transcriptomic aberrations in prostate cancer. Recent technical developments have enabled us to assess large-scale and dynamic cancer proteomes like never before. Current and future efforts, such as The National Cancer Institute’s Clinical Proteomic Tumor Analysis Consortium, collecting large-scale proteomes, glycoproteomes, and phosphoproteomes from several cancer types, including prostate cancer, will increase our understanding of the molecular basis of cancer in a similar fashion to large-scale genetic efforts in the past. In addition to a better understanding of the molecular events and targetable pathways in prostate adenocarcinoma, integrative proteogenomic profiling of NEPC is a key task for future studies, especially for understanding treatment resistance-associated tumor cell plasticity. With future improvements in instrumentation and methods for proteomics, near-complete proteomes of prostate cancer are expected soon.

## Figures and Tables

**Table 1:** MS-based, large-scale, quantitative proteomic studies of clinical prostate cancer. Studies identifying proteins in the order of thousands are listed.

Publication	Number of High-Confidence Proteins Present in All Samples	MS *** Method	Patient Samples Included in Proteomics	Additional Information	Reference
Primary prostate cancer					
Iglesias-Gato et al., 2016	4900 * (in >50% of samples)	Super-SILAC spike-in	adjacent non-malignant (n = 8), PCa (n = 28; n = 16 high-risk PCa, n = 12 low-risk PCa)		[46]
Myers et al., 2016	1500 * (in total)	label-free LC-MS/MS	matched non-malignant tissue (n = 9), PCa (n = 14)	comparing patient samples of African American and Caucasian American men	[47]
Staunton et al., 2017	2000	label-free LC-MS/MS	8 PCa (GG3 n = 4), GG4 n = 4)	Micro-dissected epithelium and stroma	[48]
Guo et al., 2018	3700	pressure cycling technology, SWATH	adjacent benign (n = 3), PCa (n = 3)	assessment of tissue heterogeneity with 3–6 spatially distinct samples/tissue type/patient (n = 60 in total)	[49]
Kawahara et al., 2019	2100 * (in total)	label-free LC-MS/MS	BPH (n = 5), PCa (n = 50; n = 10 in each ISUP grade group)		[50]
Zhou et al., 2019	3600	tandem mass tagging-SPS-MS3	adjacent normal (n = 9), PCa (n = 18; GS6 n = 9, GS8–9 n = 9)		[51]
Sinha et al., 2019	3400	label-free LC-MS/MS	localized PCa (n = 75; ISUP1 n = 4, ISUP2 n = 58, ISUP3 n = 13)	integrative with WGS (n = 74), methylome (n = 72), H3K27Ac (n = 35), transcriptome (n = 55)	[52]
Latosinska et al., 2020	1400 * (in total)	label-free LC-MS/MS	BPH (n = 5), PCa (n = 17)		[53]
Primary and advanced prostate cancer					
Liu et al., 2014	2100 ** N-glycosite peptides	SPEG for N-linked glycopeptides; SWATH-MS	non-malignant (n = 10), PCa (n = 40; non-aggressive n = 24, aggressive n = 16)	large-scale proteomics performed on pooled samples of each sample group	[54]
Drake et al., 2016	8300 ** phosphor-ylated peptides	IP for pS/T and pY phospho-proteomics, LC-MS/MS	treatment-naïve PCa (n = 11), metastatic CRPC (n = 16)	integrative with genomic and transcriptomic data	[55]
Latonen et al., 2018	3400	SWATH-MS	BPH (n = 10), PCa (n = 17), CRPC (n = 11)	integrative with WGS, methylome, and transcriptome data	[56]
Müller et al., 2018	1200 * (present in at least four cases of each sample type)	label-free LC/MS-MS	PCa (n = 5), metastasizing PCa (n = 5), LNM (n = 5)	non-metastasizing vs. metastasizing primary tumors; primary tumors vs. matched LNM	[57]
Iglesias-Gato et al., 2018	5000 *(per sample, on average)	Super-SILAC spike-in	bone distal metastases (n = 22, of which 16 CRPC)	transcriptome for certain samples; compared with non-malignant and PCa reported earlier (Iglesias-Gato et al., 2016)	[58]
Kwon et al., 2020	1600 * (in total)	TMT-LC-MS/MS	non-malignant (n = 10), PCa (n = 8), metastatic PCa (n = 2)	cancer samples according to T-stage (T2 n = 4, T3 n = 4, T3-N1 n = 2)	[59]

* Number of identified proteins as information is available, not required to be present in all samples in the dataset. In total, present in any number of the samples. ** Number of identified modified peptides. *** For non-abbreviated names and details of individual methods, the reader is referred to the original articles. BPH, benign prostatic hyperplasia; CRPC, castration-resistant prostate cancer; LNM, lymph node metastasis; MS, mass spectrometry; PCa, primary prostate cancer; WGS, whole genome sequencing.

**Table 2:** MS-based, large-scale, quantitative proteomic studies utilizing models of prostate cancer.

Publication	Models Included	Specifications	References
Cima et al., 2011	Pten−/− mice	glycoproteomes of wt and Pten−/− prostates	[90]
Tan et al., 2014	VCaP cell line	proteomic changes with downregulation of ERG with siRNA; phosphoproteomics; in comparison with clinical samples	[60]
Jiang et al., 2015	LNCaP xenografts	phosphoproteomes in intact and castrated mice	[91]
Drake et al., 2016	cell lines and xenografts (22Rv1, LNCaP)	phosphoproteomics, integrative with genomic and transcriptomic data, in comparison with clinical tumor samples (see Table 1)	[55]
Höti et al., 2017	LNCaP, LNCaP-95	proteomic changes in androgen resistance	[92]
Zhang S. et al., 2018	cell lines PC-3M-2B4, PC-3M-1E8	proteomes associated with different metastatic ability	[93]
Zhang Y. et al., 2018	TRAMP mice	proteomes of wt and TRAMP prostates	[94]
Katsogiannou et al., 2019	cell lines PNT1A8, LNCaP, DU145, PC-3	differences in proteins and phosphosites between cell lines	[95]
Kwon et al., 2019	cell lines LNCaP, PC-3 LCaP-LN3, PC-3M	comparison of parental to more metastatic sub-lines	[96]
Miao et al., 2019	cell lines PC-3, PC-3MLN4	kinome associated with increased metastasis	[97]
Singh and Sharma, 2020	cell lines PC-3, LNCaP	proteomic changes in TGF-ß-induced EMT	[98]
Zhang et al., 2020	Pten−/− mice	proteomes of wt and Pten−/− prostates	[99]
Liynage et al., 2021	cell lines LNCaP, C4-2B	proteomic changes stimulated by anti-androgens bicalutamide and enzalutamide	[100]

BPH, benign prostatic hyperplasia; CRPC, castration-resistant prostate cancer; LNM, lymph node metastasis; MS, mass spectrometry; PCa, primary prostate cancer; WGS, whole genome sequencing.

## Abbreviations

2-DE: 2-dimensional gel electrophoresis; AD, androgen-dependent; ADT, androgen deprivation therapy; AI androgen-independent; AR, androgen receptor; BPH, benign prostatic hyperplasia; CAF, cancer-associated fibroblast; CRPC, castration-resistant prostate cancer; DEP, differentially expressed protein; DHT, Dihydrotestosterone; ECM, extracellular matrix; EMT, epithelial to mesenchymal transition; FFPE, formalin-fixed, paraffin-embedded; GS, Gleason score; IHC, immunohistochemistry; ISUP, International Society of Urological Pathology; LCM, laser-capture micro-dissection; LNM, lymph node metastases; MS, mass spectrometry; MSI, mass spectrometry imaging; NE, neuroendocrine; NEPC, neuroendocrine prostate cancer; NPF, non-malignant prostate fibroblasts; PCa, primary prostate cancer; PC, prostate cancer; PDX, patient-derived xenograft; PSA, prostate-specific antigen; RIME, rapid immunoprecipitation MS of endogenous proteins; SILAC, Stable Isotope Labeling by/with Amino acids in Cell culture; TCA, tricarboxylic acid cycle; wt, wild-type.
